# Identification of altered metabolic functional components using metabolomics to analyze the different ages of fruiting bodies of *Sanghuangporus vaninii* cultivated on cut log substrates

**DOI:** 10.3389/fnut.2023.1197998

**Published:** 2023-08-03

**Authors:** Congtao Xu, Shuang Zhao, Zihao Li, Jinlong Pan, Yuanyuan Zhou, Qingxiu Hu, Yajie Zou

**Affiliations:** ^1^Institute of Agricultural Resources and Regional Planning, Chinese Academy of Agricultural Sciences, Beijing, China; ^2^Institute of Agri-Food Processing and Nutrition, Beijing Academy of Agriculture and Forestry Sciences, Beijing, China

**Keywords:** *Sanghuangporus vaninii*, metabolomics, growth age, LC-MS, functional components

## Abstract

*Sanghuangporus vaninii* is a profitable traditional and medicinal edible fungus with uncommon therapeutic properties and medicinal value. The accumulation of active ingredients in this fungus that is used in traditional Chinese medicine is affected by its years of growth, and their pharmacological activities are also affected. However, the effects of age on the medicinal value of fruiting bodies of *S. vaninii* cultivated on cut log substrate remain unclear. In this study, an untargeted liquid chromatography mass spectrometry (LC-MS)-based metabolomics approach was performed to characterize the profiles of metabolites from 1-, 2- and 3-year-old fruiting bodies of *S. vaninii*. A total of, 156 differentially accumulated metabolites (DAMs) were screened based on the criterion of a variable importance projection greater than 1.0 and *p* < 0.01, including 75% up regulated and 25% down regulated. The results of enrichment of metabolic pathways showed that the metabolites involved the biosynthesis of plant secondary metabolites, biosynthesis of amino acids, central carbon metabolism in cancer, steroid hormone biosynthesis, linoleic acid metabolism, prolactin signaling pathway, and arginine biosynthesis, and so on. The biosynthesis of plant secondary metabolites pathway was significantly activated. Five metabolites were significantly elevated within the growth of fruiting bodies, including 15-keto-prostaglandin F2a, (4S, 5R)-4,5,6-trihydroxy-2-iminohexanoate, adenylsuccinic acid, piplartine, and chenodeoxycholic acid. 15-keto-prostaglandin F2a is related to the pathway of arachidonic acid metabolism and was significantly increased up to 1,320- and 535-fold in the 2- and 3-year-old fruiting bodies, respectively, compared with those in the 1-year-old group. The presence of these bioactive natural products in *S. vaninii* is consistent with the traditional use of Sanghuang, which prompted an exploration of its use as a source of natural prostaglandin in the form of foods and nutraceuticals. These findings may provide insight into the functional components of *S. vaninii* to develop therapeutic strategies.

## Introduction

Sanghuang is a famous traditional edible and medicinal fungus and has been recognized as beneficial to health for more than 2,000 years. Its medical value was first recorded in the Chinese medicine monograph “Shennong materia medica classic” and was initially named “mulberry ear” (1). Modern pharmacological research has demonstrated that Sanghuang possesses various remarkable biological activities, such as anti-inflammatory (2), liver-protecting (3), anti-tumor (4), hemostatic (5), hypoglycemic, and immunomodulatory effects (6). It is valuable in traditional Chinese medicine and widely used in East Asia, including China, Japan, and Korea. According to the latest classification in 2016, a total of 15 species are currently found owing to the different species of parasitic trees. *Sanghuangporus vaninii* only lives on the dead fallen or vertically living poplar (*Populus* spp.) trees, and is distributed in the forested areas of northeast China, as well as Korea, far eastern Russia, Japan, and the United States (1). As the consumer demand for Sanghuang products has gradually increased, the fruiting bodies of *S. vaninii* are primarily obtained by artificial cultivation (2). The common technology that is used to cultivate *S. vaninii* includes substitute and cut log cultivation. Previous studies have shown that *S. vaninii* is highly valuable medicinally and has abundant active constituents including polysaccharides (7), polyphenolics, flavonoids, and terpenoids among others, and these bioactive components endow *S. vaninii* with antioxidant (8), antitumor, and hypoglycemic activities (9). Therefore, most of the research on *S. vaninii* is focused on the extraction of polysaccharides, optimization of deep fermentation conditions, molecular structure, and anti-cancer immune mechanisms. However, this research is still in its infancy. Determining how to improve the yield of *S. vaninii* and its bioactive substances is still an area of highly active study by researchers.

Furthermore, the intricate modifications in the active constituents within organisms present a challenge in unraveling their complex activities. Metabolomics, as a current and innovative technology (10), has the potential to furnish extensive metabolic information for multiple samples, which encompasses metabolic pathways, metabolites, and their levels of relative expression (11, 12). An amalgamation of bioinformatics analytics can facilitate the exploration of key metabolic processes and biological pathways that are associated with the targeted disease, thereby ascertaining the potential medicinal value of *S. vaninii*. Metabolomics offers high resolution, which enables the differentiation of metabolic differences among diverse compounds and a better comprehension of their mechanisms of action (13). In addition, it is highly reproducible, which guarantees the dependability of its detection outcomes and promotes data sharing and comparison. The identification of compounds that possess regulatory effects on metabolism and the elucidation of their role in the body are of immense importance, as evidenced in humans, animals, plants, and microorganisms, and relevant exogenous/endogenous disturbances are also being observed on a large scale (11, 12, 14, 15).

In recent years, the use of metabolomics in the research of edible fungi has been extensively applied, and the application of metabolomics to the study of bioactive substances in edible fungi have been identified (16). *Ganoderma lucidum* is a traditional medicinal edible fungus owing to its primary active ingredients, which exhibit various biological activities, such as antioxidation, anti-virus, and anti-tumor activities. Gas chromatography-mass spectrometry (GC-MS) and liquid chromatography-mass spectrometry (LC-MS) has been used to study the mechanism that underlies the morphology and effect of methyl jasmonate (MeJA) on the metabolism of *G. lucidum* (17, 18). Analyses that utilized gas chromatography-time-of-flight-mass spectrometry (GC-TOF-MS) and ultra-high performance liquid chromatography tandem mass spectrometry (UHPLC-MS/MS) have shown that metabolomic variations in the spatial (cap and stipe) influence the functional composition between brown and white beech mushrooms (*Hypsizygus marmoreus*). The cap has a high content of amino acids that control the taste and nutrition of the mushrooms, and the brown strain contains a large amount of hypsiprenol (19). An approach to metabolomics that was based on untargeted ultra-high performance liquid chromatography quadrupole time-of-flight mass spectrometry (UPLC-Q-TOF-MS) was used to explore the metabolite profiles of morel mushrooms (*Morchella* spp.) from four distinct geographical origins of China and pioneered high-throughput methodology to evaluate the quality of species of *Morchella* (12). Mohamed (20) used GC-MS to analyze the heterogeneity of their metabolites in the context of the volatile profile of sensory and nutrients of the truffles *Terfezia claveryi* and *T. boudieri*. *T. claveryi* provided a better composition of sugar in the diet owing to its production of fewer sugars and higher levels of sugar alcohols compared with that of *T. boudieri* (20). It was found that the 3-year old fruiting body had the best anti-tumor effects. We concluded that the metabolic regulatory pathway of *S. vaninii* fruiting bodies must change during their growth, and that a metabolomics approach would screen out some significant increased metabolites that may be potential indicators for functional components.

A metabolomics approach that utilized UPLC-Q-TOF-MS was applied to investigate the physiochemical distinctions among the 1-, 2-, and 3-year-old fruiting bodies of *S. vaninii.* The main aim of this study was to elucidate the metabolic profiles of samples from various age groups, which would aid in the exploration of the medicinal and economic values of this organism. In addition, the outcomes of this study could be utilized to differentiate the origins of the fruiting bodies based on their age. The findings of this study should serve as evidence for the quality control and further development of products made from *Sanghuangporus* products.

## Materials and methods

### *Sanghuangporus vaninii* cultivation

Strain QF-3 of *S. vaninii* was provided by the Zhejiang Qianji Fang Pharmaceutical Technology Co., Ltd. (Hangzhou, China). It was stored in the China General Microbiological Culture Collection Center, and the conservation number is CGMCC No.20227. The mycelia were activated on potato dextrose agar (PDA) media before use. The cultivation substrate was prepared from cut logs. Each section was cut into 3–4 pieces and then formed into a 15–17 cm diameter wooden bale (3–4.5 kg), tied with plastic rope, and soaked in water for 20 min. The water was then removed. The water-controlled wooden bundle was placed into the polypropylene bags that were 22 cm wide and 45 cm long, or both ends of the wooden section were filled with a 3:1 layer of wooden sawdust and wheat bran nutritional material (v/v). An appropriate amount of water was added and mixed well, and the water content was 64–66%. The bag opening was tied, and the wood and the plastic bag were closely fitted. The substrates were sterilized for 3.5 h, and cooled to room temperature. The inoculated cut logs were kept at 25–28°C in the dark with a relative humidity of 60–65% to facilitate mycelial growth. The indoor air was kept fresh by shading culture and proper ventilation. When the pale yellow mycelia fully covered the substrates within 45–60 days, the culture bags were transferred to the mushroom house at 28–30°C and 80–90% relative humidity to induce the differentiation and development of the fruiting body. After 1-, 2- and 3-year-old fruiting bodies were harvested, they were cut into 2–4 mm thick pieces and dried at 60°C in an oven to maintain a constant weight. All samples were ground to powder and stored in a sealed container until the metabolites were analyzed.

### Extraction of metabolites from *S. vaninii*

An appropriate amount of the sample was treated with 50% of aqueous methanol and redissolved with a 4-ppm solution of 2-amino-3-(2-chloro-phenyl)-propionic acid to obtain the filtrate for LC-MS detection. The metabolites were extracted as previously described (21). A volume of 20 μL from each prepared sample was used for the quality control samples (QC) and quality assurance samples (QA) to correct the deviation of the results and the errors caused by the analytical instrument itself.

Liquid chromatography analysis was conducted on Vanquish UHPLC (Thermo Fisher Science Company, Waltham, MA, USA). An ACQUITY HSS T3 UPLC^®^ column (150 × 2.1 mm, 1.8 microns) (Waters, Milford, MA, USA) was used for the chromatographic analysis. The column temperature was maintained at 40°C; the sample flow rate was 0.25 mL min^−1^, and the sample volume was 2 μL. The gradient for elution utilized 0.1% formic acid-acetonitrile (C) and 0.1% formic acid-water (D), and the compounds that eluted were identified by liquid chromatography-mass spectrometry (LC-ESI (+)-MS). A sample of 2% carbohydrates was applied at 0–1 min. The C was between 2 and 50% from 1 to 9 min. When the separation phase was between 9 and 12 min, the C value was between 50 and 98%. The value when the separation phase was between 12 and 13.5 min was 98%. The separation phase range was 13.5–14 min, and the range of C values was from 98 to 2%. The separation phase was between 14 and 20 min, and the C value was 2%. In LC-ESI (-)-mass spectrometry, the gradient components were (A) acetonitrile and (B) 5 mM ammonium formate. The sample was separated under conditions of 0-1 min and 2% A. At 1–9 min, there was between 2 and 50% A. At 9–12 min, there was between 50 and 98% A. At 12–13.5 min, the A value was 98%, which was the highest value. At 13.5–14 min, the A value was between 98% and 2%. At 14–17 min, 2% A was appropriate (22).

Electrospray ionization tandem mass spectrometry was tested on an Orbitrap Exploris 120 (Thermo Fisher Scientific) to detect the mass spectra of metabolites. These parameters were as follows: cover pressure, 30 mbar. Auxiliary gas flow, 10 bar. The discharge potential of ESI (+) was 3.50 kV, and the discharge potential of ESI (-) was 2.50 kV, 325°C. MS1 was between 100 and 1000. Resolution of MS1, hologram of 60000 FWHM. The data correlation in each loop was 4; The resolution of mass spectrometry was 15000 FWHM; Standard collision energy, 30%; and an automatic dynamic clearance time (23).

### Statistical analysis

To be able to utilize the features and align the retention time, the raw data were initially transformed into the mzXML format using MS Convert from the Proteo Wizard software suite (v3.0.8789). The MS/MS data were matched with HMDB, MassBank, LIPID MAPS, mzCloud, and the Kyoto Encyclopedia of Genes and Genomes (KEGG) to accurately identify the metabolites with accuracy mass (< 30 ppm). The data were normalized to compensate for any systematic bias using the robust LOESS signal correction (QC-RLSC). Only the ion peaks whose relative standard deviation (RSD) in the QC was less than 30% were retained after normalization, in order to ensure that the correct metabolite was identified.

The R software package RoPLS was used to conduct a principal component analysis (PCA) and an orthogonal projections to latent structures discriminant analysis (OPLS-DA). The PCA method was utilized to establish patterns of intra-group clustering and inter-group segregation, while the OPLS-DA was employed to delve deeper into the distinctions in the inter-group. The data were scaled and the score plot, load plot, and sPlot plot were plotted to show the difference in composition of metabolites between the samples. Through the permutation test for overfitting, the suitability of the method is verified. OPLS-DA can use variable importance variability prediction projection (VIP) to identify differential metabolites. Through the *P*-value and VIP information obtained by the combination of OPLS-DA and follow-folding (FC), the key factors affecting the classification results are found out. *P*-value < 0.05 and VIP values > 1 indicated that there were significant differences in the content of metabolites. The pathways of different metabolites were analyzed by Metbra Analyst 4.0 software, and the pathway topology was integrated with the method of pathway analysis. On this basis, through the analysis of the KEGG pathway, the physiological function of the pathway was revealed at a higher level. The metabolites and corresponding pathways were visualized by KEGG drawing software.

## Results

### Effect of different years on the morphological properties of *S. vaninii*

**FIGURE 1:**
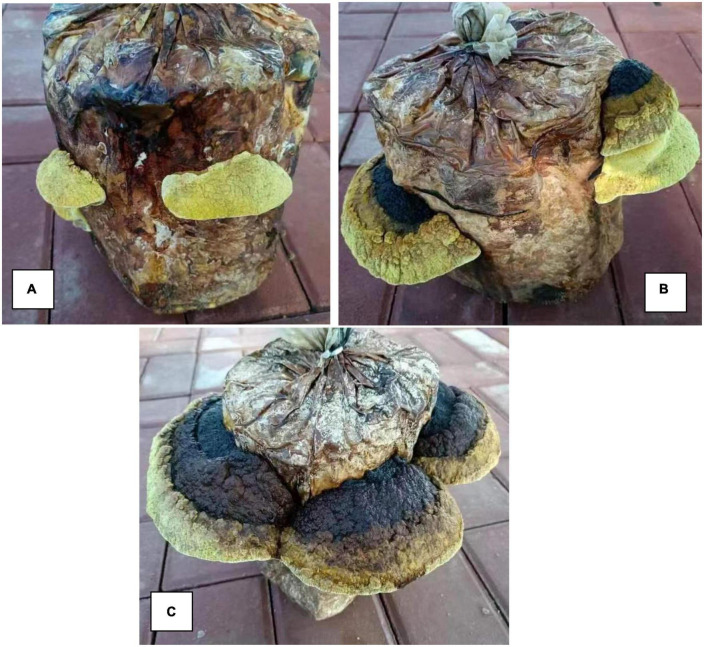
Morphological characteristics of *Sanghuangporus vaninii* fruiting bodies from the different years of growth. **(A)** The 1-year-old fruiting bodies (Group A), **(B)** the 2-year-old fruiting bodies (Group B), **(C)** the 3-year-old fruiting bodies (Group C).

Figure 1 shows the morphological properties of the *S. vaninii* fruiting body grown for different numbers of years. The shape of the 1-year-old fruiting body was regular, which was a horseshoe, fan, or shell shape without clear rings and completely yellow. The 2-year-old fruiting body was regularly shaped, which was a horseshoe, fan, or shell shape with clear rings. The front side was blackish brown or brown to brownish yellow. The shape of 3-year-old fruiting body was regular, which was a horseshoe, fan, or shell shape with clear rings. The front side was blackish brown or brown to brownish yellow.

### LC-MS ion monitoring analysis

**FIGURE 2:**
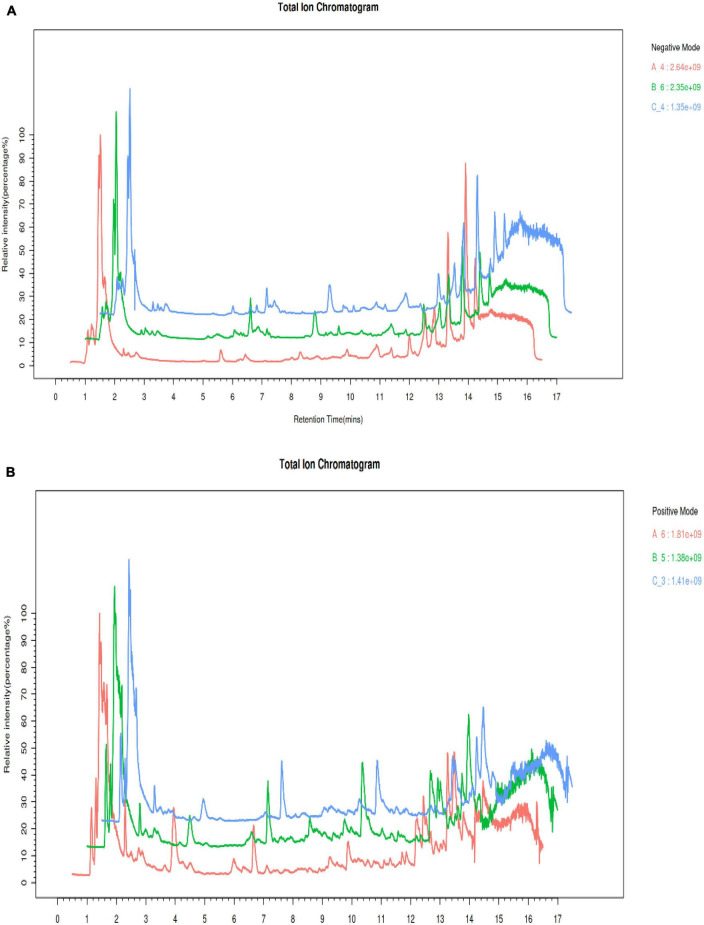
Comparison of the total ion chromatogram (TIC) under electrospray ionization (ESI) **(A)** positive mode. **(B)** Negative mode.

The present investigation utilized an LC-MS methodology to explore the variations of metabolites in *S. vaninii* fruiting bodies over different growth years. In particular, the extracellular fluid from fruiting bodies of 1-year-old (Group A), 2-year-old (Group B), and 3-year-old (Group C) was subjected to analysis. The LC-MS total ion current (TIC) chromatograms that were obtained from Groups A, B, and C are depicted in Figure 2. The findings revealed that the specimens examined using this approach displayed robust signal strength, high peak capacity, favorable retention time, and strong reproducibility.

### Principal component analysis

**FIGURE 3:**
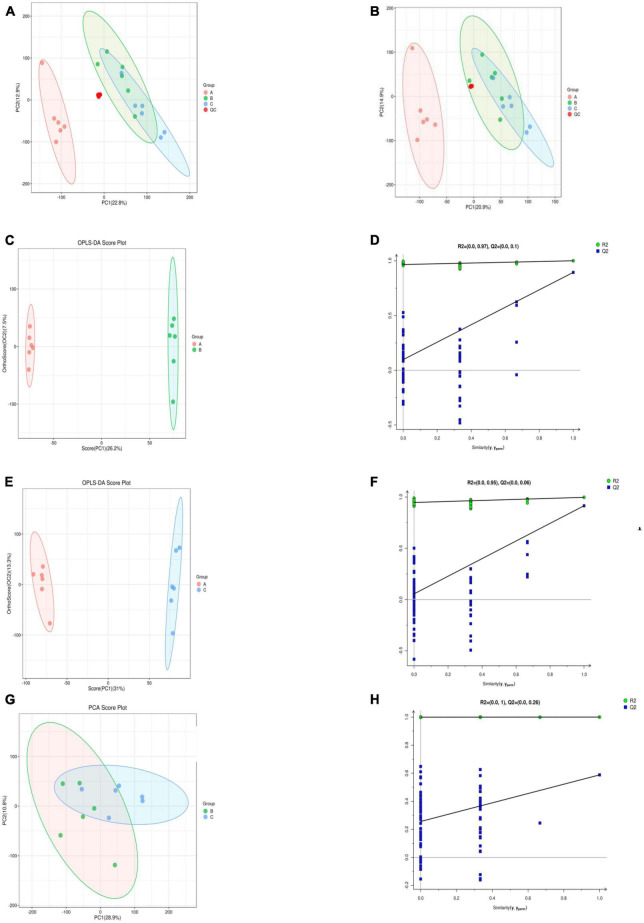
Supervised and unsupervised multivariate analysis based on the metabolomic data of fruiting bodies. **(A)** Principal component analysis in the positive ion mode. **(B)** Principal component analysis in the negative ion mode. **(C,D)** OPLS-DA scores plot and permutation testing between Group A and B. **(E,F)** OPLS-DA scores plot and permutation testing between Group A and C. **(G,H)** OPLS-DA scores plot and permutation testing between Group B and C. OPLS-DA, orthogonal projections to latent structures discriminant analysis. Group A, 1-year-old fruiting bodies; Group B, 2-year-old fruiting bodies; and Group C, 3-year-old fruiting bodies.

An unsupervised PCA analysis method was used to distribute all the samples as a whole and stabilize the overall analytical procedure. The distribution of QC samples was dense when the metabolites were detected with LC-MS, indicating that the detection was highly stable and reliable. A QA was performed based on a QC to facilitate detection of the biomarker, the characteristic peaks that did reproduce well were deleted from the QC sample, and the proportion of characteristic peaks (RSD < 30%) in the samples was 86.6% for the positive mode and 85.1% for the negative mode; this showed that the data were good and repeatable. The coordinates of all the samples were within the 95% confidence interval, which indicated that the model could be used to analyze the differences of *S. vaninii* metabolites at different growth stages. The distribution of groups A, B and C was situated on the negative *X*-axis, near the origin, and on the positive *X*-axis, respectively, indicating that the metabolites of *S. vaninii* had changed significantly over the years of growth in Figures 3A, B. Samples of the 1-year-old fruiting bodies were significantly separated from the others, which highlighted the difference in metabolites among the years of growth. The results partially overlapped in the 2-year-old fruiting bodies (Group B) and 3-year-old fruiting bodies of *S. vaninii*, which could have resulted from the similar metabolites and their contents during these two years of growth. Six replicates of each year clustered together, indicating that most of the data were densely distributed and highly repeatable.

### Partial least-squares discriminant analysis

The group separation was further adjusted using the supervised OPLS-DA model in order to confirm the differences in metabolism between the three groups of fruiting body samples. Fruiting bodies samples A, B, and C were compared pairwise using OPLS-DA, and the results showed that there were significant metabolic differences existed among the classes in the first part of each pairwise comparison (Figures 3C, E, G). For the OPLS-DA model, the values of R2Y and Q2 are all above 0.5 (Table 1), which shows that the model fits well and the prediction performance is good. Therefore, the OPLS-DA model is suitable for analyzing the differences among the three research objects. To further verify the reliability and stability of the OPLS-DA model and whether there was an over-fitting phenomenon, 200-time iterative permutation tests were used. All the Q2 points in A vs. B and A vs. C were lower than the original Q2 points that were situated on the right. The R2-intercepts were 0.97 and 0.95, and the Q2-intercepts were 0.1 and 0.06, respectively (Figures 3D, F). The results demonstrated that the OPLS-DA model is acceptable and does not overfit. However, there was an overfitting between B vs. C (Figure 3H), which could be owing to the non-significant difference in metabolites between the two groups. This result corresponded to the PCA score plot in Figure 2. In general, the data can be used to screen the different metabolic products of the fruiting body with the differing ages of growth.

**TABLE 1:** Validation parameter of the OPLS-DA model.

Comparison	Pre	R2X (cum)	R2Y (cum)	Q2 (cum)
A vs. B	1 + 1 + 0	0.337	1.0	0.894
A vs. C	1 + 1 + 0	0.443	0.997	0.915
B vs. C	1 + 1 + 0	0.314	0.989	0.858
A vs. B vs. C	1 + 2 + 0	0.466	1.0	0.588

OPLS-DA, orthogonal projections to latent structures discriminant analysis. A, 1-year-old fruiting bodies; B, 2-year-old fruiting bodies; and C, 3-three-year old fruiting bodies.

Based on OPLS-DA’s findings, the metabolites with significant differences were screened by VIP values > 1.0 and *P* < 0.05 (two-tailed Student’s *t*-test). Because the same metabolite can differ significantly in different groups, 248 differentially abundant metabolites (DAMs) in the fruiting bodies of *S. vaninii* were identified between the three ages of growth (Supplementary Table 1). They included 12 alcohols; 5 alkaloids and derivatives; 38 amino acids, peptides, and analogs; 18 benzenoids; 25 carbohydrates and carbohydrate conjugates; 4 fatty acid esters; 18 fatty acids and conjugates; 3 indoles and derivatives; 1 inorganic compound; 2 isoflavonoids; 6 lactones; 5 types of linoleic acids and derivatives; 15 lipids and lipid-like molecules; 26 nucleosides, nucleotides, and analogs; 16 organic acids and derivatives; 11 organoheterocyclic compounds; 1 organonitrogen; 5 organooxygen compounds; 10 phenolics; 18 steroids and steroid derivatives; and 9 terpenoids (Table 2). Several metabolites were found infrequently in the fruiting bodies collected at 1 year (A sample) but they markedly increased in the fruiting bodies collected at 2 and 3 years (B and C samples), including 15-keto-prostaglandin F2α, xanthine and (4S, 5R)-4,5,6-trihydroxy-2-iminohexanoate. To facilitate the analysis, 156 DAMs were screened out based on the criterion of VIP greater than 1.0 and *P* < 0.01 and listed in Table 3. The fold-changes (FC) for each metabolite between each two samples are also listed.

**TABLE 2:** Types of compounds and changes of different metabolites among 1-, 2- and 3-year-old fruiting bodies of *S. vaninii*.

No.	Species of metabolites	A vs. B			A vs. C			B vs. C		
		Total	Up	Down	Total	Up	Down	Total	Up	Down
1	Terpenoids	5	3	2	5	5		4	4	
2	Steroids and steroid derivatives	14	6	8	11	6	5	5	5	
3	Phenolics	9	4	5	7	5	2	3	3	
4	Organooxygen compounds	3	3		5	4	1	1	1	
5	Organonitrogen compounds	1	1		1	1		1	1	
6	Organoheterocyclic compounds	6	4	2	9	7	2	3	3	
7	Organic acids and derivatives	14	9	5	14	12	2	3	3	
8	Nucleosides, nucleotides, and analogs	16	7	9	18	10	8	12	10	2
9	Lipids and lipid-like molecules	13	8	5	10	8	2	5	5	
10	Lactones	3	2	1	5	2	3	3	2	1
11	Isoflavonoids	2	1	1	2	1	1			
12	Inorganic compounds				1	1		1	1	
13	Indoles and derivatives	2	2	0	3	3		3	3	
14	Fatty acids and conjugates	10	8	2	21	20	1	7	7	
15	Fatty acid esters	3	1	2	2	2		1	1	
16	Carbohydrates and carbohydrate conjugates	8	5	3	19	15	4	12	10	2
17	Benzenoids	12	8	4	14	11	3	6	6	
18	Amino acids, peptides, and analogs	25	16	9	30	21	9	13	13	
19	Alkaloids and derivatives	3	1	2	4	3	1			
20	Alcohols and polyols	6	4	2	10	9	1	5	5	

**TABLE 3:** Metabolic pathways and significant different metabolites that are enriched in these pathways between different groups.

Group	Pathway-name	Metabolites	*P*-value	Pathway-ID
A vs. B	Biosynthesis of plant secondary metabolites	AMP; L-glutamic acid; L-arginine; L-serine; L-tyrosine; GMP; L-malic acid; nicotinic acid; prephenate; L-DOPA; xanthine; shikimic acid; paraxanthine	4.59 × 10^–5^	map01060
	Biosynthesis of amino acids	L-glutamic acid; L-arginine; L-serine; L-tyrosine; ketoleucine; prephenate; saccharopine; shikimic acid; N-acetylglutamic acid; diaminopimelic acid; homocitric acid; (R)-2,3-dihydroxy-isovalerate	7.72 × 10^–5^	map01230
	Steroid hormone biosynthesis	progesterone; androsterone; cortisol; corticosterone; 17α-estradiol; 11β-hydroxyandrost-4-ene-3,17-dione; adrenosterone; 7α-hydroxyandrost-4-ene-3,17-dione; 2-methoxy-17β-estradiol; 11β,17a,21-trihydroxypregnenolone	1.6 × 10^–4^	map00140
	Cocaine addiction	L-glutamic acid; L-tyrosine; L-DOPA	5.01 × 10^–4^	map05030
	Taste transduction	AMP; L-glutamic acid; GMP; L-malic acid; γ-aminobutyric acid	1.07 × 10^–3^	map04742
A vs. C	Biosynthesis of plant secondary metabolites	AMP; L-glutamic acid; L-arginine; L-serine; Ornithine; L-tyrosine; L-malic acid; L-asparagine; L-threonine; nicotinic acid; prephenate; L-DOPA; xanthine; tyramine; shikimic acid; α-Linolenic acid; paraxanthine	1.27 × 10^–6^	map01060
	Biosynthesis of amino acids	L-glutamic acid; L-arginine; L-serine; ornithine; L-tyrosine; L-asparagine; L-threonine; ketoleucine; prephenate; oxoadipic acid; saccharopine; shikimic acid; N-acetylglutamic acid; diaminopimelic acid; homocitric acid; (R)-2,3-dihydroxy-isovalerate	1.62 × 10^–6^	map01230
	Central carbon metabolism in cancer	L-glutamic acid; L-arginine; L-serine; L-tyrosine; glucose 6-phosphate; L-malic acid; L-asparagine	1.12 × 10^–4^	map05230
	Linoleic acid metabolism	13-L-hydroperoxylinoleic acid; γ-Linolenic acid; 13-OxoODE; 9-OxoODE; alpha-dimorphecolic acid; 9,10-12,13-diepoxyoctadecanoate	1.7 × 10^–4^	map00591
	Prolactin signaling pathway	L-tyrosine; glucose 6-phosphate; L-DOPA; Progesterone	2.45 × 10^–4^	map04917
B vs. C	ABC transporters	L-aspartic acid; L-arginine; L-serine; choline; inosine; uridine; deoxyguanosine; D-xylitol; guanosine; mannitol; cytidine	3.13 × 10^–6^	map02010
	Arginine biosynthesis	L-aspartic acid; L-arginine; N-acetylglutamic acid; argininosuccinic acid	2.75 × 10^–4^	map00220
	Biosynthesis of amino acids	L-aspartic acid; L-arginine; L-serine; ketoleucine; N-acetylglutamic acid; homocitric acid; argininosuccinic acid; N-Acetyl-L-2-amino-6-oxopimelate	4.32 × 10^–4^	map01230
	Taste transduction	GMP; L-malic acid; γ-aminobutyric acid; cyclic, AMP	1.02 × 10^–3^	map04742
	Central carbon metabolism in cancer	L-aspartic acid; L-arginine; L-serine; L-malic acid	1.76 × 10^–3^	map05230

### Hierarchical cluster analysis

**FIGURE 4:**
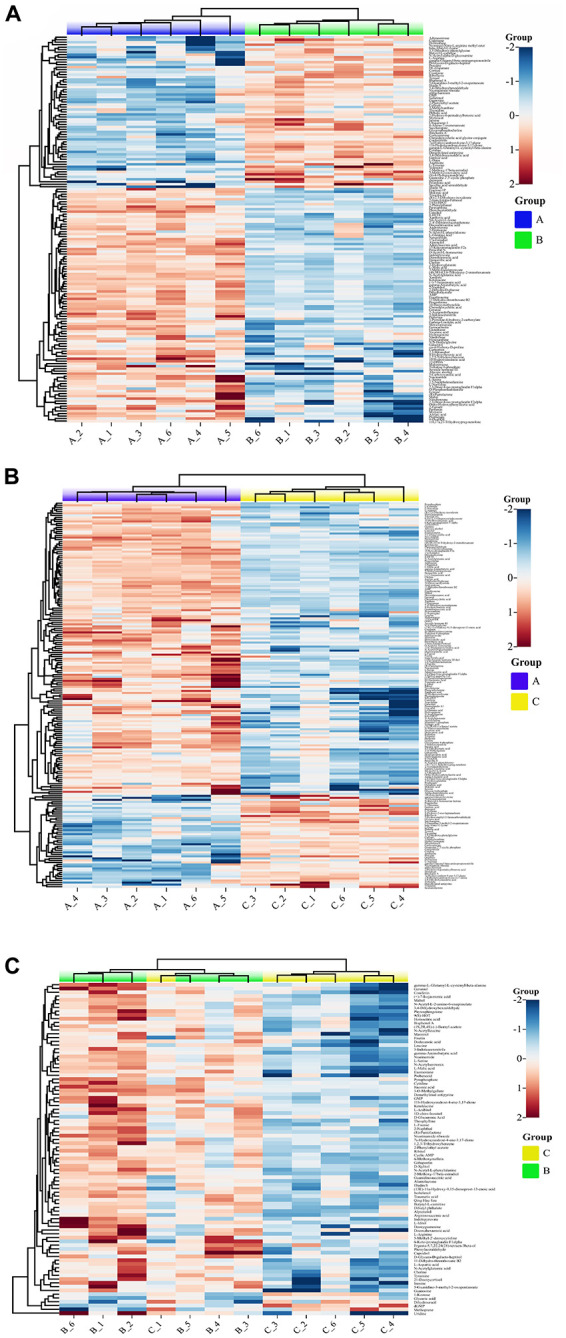
Differential metabolite volcano map (*p* < 0.01). **(A)** Group A and B, **(B)** Group A and C, **(C)** Group B and C (*p* < 0.01). Group A, 1-year-old fruiting bodies; Group B, 2-year-old fruiting bodies; and Group C, 3-year-old fruiting bodies.

**FIGURE 5:**
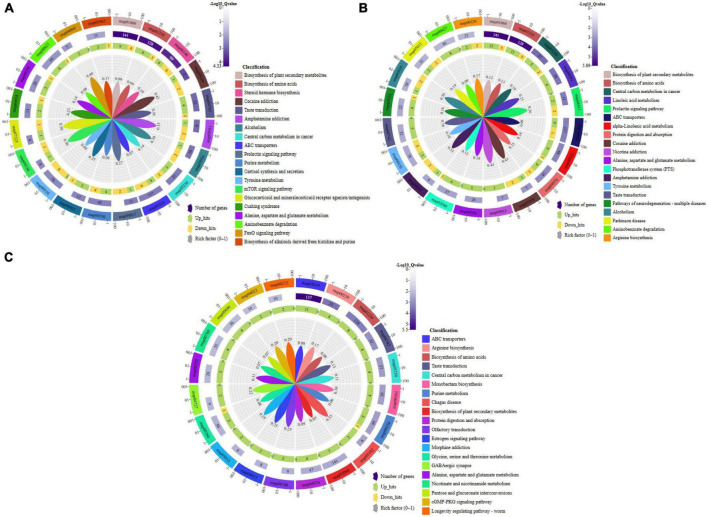
Pathway enrichment analysis of the differential metabolites: **(A)** Group A and B, **(B)** Group A and C, **(C)** Group B and C. Group A, 1-year-old fruiting bodies; Group B, 2-year-old fruiting bodies; Group C, 3-year-old fruiting bodies.

Approximately 156 identified metabolites exhibited significant dynamic variations, among samples A, B, and C from the fruiting bodies when a heat map analysis was performed (Figures 5A–C). Compared with the samples A, the metabolites that significantly decreased or increased in samples B and C were clearly separated. Significantly increased metabolites in B and C compared with the A samples were clearly observed in the upper part of Figure 4. While the upper part of this figure consists of significantly up regulated metabolites in samples B and C compared with samples A, including 15-keto-prostaglandin F2α, xanthine, N-acetylglutamic acid, ketoleucine, L-malic acid, and pyrophosphate. Figure 4C shows the differential metabolites in samples C vs. samples B. Metabolites that significantly increased in samples C compared with samples B consisted of butyryl-L-carnitine, (13E)-11α-hydroxy-9,15-dinoprost-13-enoic acid, N-acetylserotonin, maltol, and alantolactone among others. In contrast, significantly decreased metabolites included dGMP, methoprene, D-dihydrouracil, 1-kestose and glyceric acid. Figure 5 shows the correlation analysis of differential metabolites between samples A and B (A), samples A and samples C (B), and samples B and C (C). Larger values demonstrate a higher correlation between the two metabolites.

### KEGG pathways enriched by the DAMs

The metabolic differences among the three groups were summarized. A total of 156 differential metabolites that were identified were submitted to the KEGG database for an analysis of the enrichment of metabolic pathway. The significant differences in metabolic pathways among the different components and the metabolites involved are shown in Figure 5. There were 189 metabolic pathways in group B compare with group A, and 19 of them were significantly different (*P* ≤ 0.01) (Figure 5A). To facilitate the analysis, only the five most significant metabolic pathways are shown owing to the excessive number of metabolic pathways, and the analysis of the other components described below was the same. They included map01060 (Biosynthesis of plant secondary metabolites), which was composed of 13 metabolic components, including AMP, L-glutamic acid, L-arginine, L-serine, L-tyrosine, GMP, and L-malic acid among others; map01230 (Biosynthesis of amino acids), which was composed of 12 metabolic components, including L-glutamic acid, L-arginine, L-serine, L-tyrosine, ketoleucine, prephenate, and saccharopine among others; map00140 (Steroid hormone biosynthesis), which was composed of 10 metabolic components, including progesterone, androsterone, cortisol, corticosterone, 17α-estradiol, 11β-hydroxyandrost-4-ene-3,17-dione, adrenosterone, and 7α-hydroxyandrost-4-ene-3,17-dione among others; map05030 (Cocaine addiction), which was composed of three metabolic components, including L-glutamic acid, L-tyrosine, and L-DOPA; map04742 (Taste transduction), which was composed of five metabolic components, including AMP, L-glutamic acid, GMP, L-malic acid, and γ-aminobutyric acid. There were 206 metabolic pathways in group C compare with group A, and 29 of them were significantly different (*P* ≤ 0.01) (Figure 5B). They included map01060 (Biosynthesis of plant secondary metabolites), which was composed of 17 metabolic components, including AMP, L-glutamic acid, L-arginine, L-serine, and ornithine L-tyrosine among others; map01230 (Biosynthesis of amino acids), which was composed of 16 metabolic components, including L-glutamic acid, L-arginine, L-serine, ornithine, L-tyrosine, and L-asparagine among others; map05230 (Central carbon metabolism in cancer), which was composed of seven metabolic components, including L-glutamic acid, L-arginine, L-serine, L-tyrosine, glucose 6-phosphate, L-malic acid, and L-asparagine; map00591 (Linoleic acid metabolism), which was composed of six metabolic components, including 13-L-hydroperoxylinoleic acid, γ-linolenic acid, 13-oxoODE, 9-oxoODE, α-dimorphecolic acid, and 9,10-12,13-diepoxyoctadecanoate; map04917 (Prolactin signaling pathway), which was composed of four metabolic components, including L-tyrosine, glucose 6-phosphate, L-DOPA, and progesterone. There were 180 metabolic pathways in group C compare with group B. A total of 20 were significantly different (*P* ≤ 0.01) (Figure 5C). They included map02010 (ABC transporters), which was composed of 11 metabolic components, including L-aspartic acid, L-arginine, L-serine, choline, and inosine among others; map00220 (Arginine biosynthesis), which was composed of four metabolic components, including L-aspartic acid, L-arginine, N-acetylglutamic acid, and argininosuccinic acid; map01230 (Biosynthesis of amino acids), which was composed of eight metabolic components, including L-aspartic acid, L-arginine, L-serine, ketoleucine, and N-acetylglutamic acid among others; map04742 (Taste transduction), which was composed of four metabolic components, including GMP, L-malic acid, γ-aminobutyric acid, cyclic AMP, and 9,10-12,13-diepoxyoctadecanoate; map05230 (Central carbon metabolism in cancer), which was composed of four metabolic components, including L-aspartic acid, L-arginine, L-serine, and L-malic acid. These pathways mainly focused on secondary metabolic pathways.

## Discussion

In recent years, *S. vaninii* has been recognized by increasing numbers of consumers owing to its extremely high health, excellent nutritional, and medicinal values (24). The age of growth strongly influences the metabolites and quality of *S. vaninii* fruiting bodies and is an important parameter to evaluate the quality of fruiting bodies (25). It is generally accepted in China that the 3-year-old fruiting bodies of *S. vaninii* are of higher quality and more nutritious than the 1- and 2-year-old fruiting bodies. However, the differences in quality and therapeutic function among those fruiting bodies remain unclear. Therefore, the physical traits and metabolic profiles of samples were comprehensively compared at different marketable ages to investigate the factors that affect the accumulation of metabolites. As anticipated predicted, the outcomes indicated a noticeable contrast in the metabolite accumulation between the samples. Notably, they differed in the types of fatty acids and their conjugates, amino acids, peptides and analogs, nucleosides, and analogs, and steroids and steroid derivatives.

The results of the statistical analysis and the VIP value acquired from the OPLS-DA analysis collectively suggested that the 156 differential metabolites exhibited significant alterations among the three groups of fruiting bodies samples. A heat map (Figure 4A) showed that 75% of the extracellular metabolites were significantly increased in their contents, while 25% of them decreased as the growth of fruiting bodies reached their harvesting standard. The results of enrichment of metabolic pathways indicated that many were involved in various metabolic processes, such as the biosynthesis of plant secondary metabolites, amino acid metabolism, and steroid hormone biosynthesis.

Recently, the metabolic pathways and physiological functions of amino acids have attracted a wide range of attention. Amino acids are involved in a variety of physiological and dietary purposes in fungal growth and development (24, 26). Some amino acids, such as glutamic acid, arginine, and serine, can be converted to precursors of gluconeogenesis, which can provide building blocks and energy (27, 28). Certain amino acids increased in the 2- and 3-year-old fruiting bodies compared with the controls. The relative contents of glutathione (2.65-fold), N-acetylleucine (2.7-fold), N-acetyl-L-phenylalanine (2.28-fold), leucine (2.89-fold), and L-glutamic acid (2.89-fold) (*P* < 0.01) increased significantly in the 3-year-old group. The relative contents of N,N-diethylglycine (1.76-fold) increased significantly (*P* < 0.01) in the 2-year-old group. The abundance of N6-acetyl-L-lysine showed the most significant changes (*P* < 0.01) that accompanied the growth of fruiting bodies. N6-acetyl-L-lysine is a post-translationally modified version of lysine, indicating that the lysine degradation pathway might be activated to promote the biosynthesis of acetoacetyl-CoA. Lysine shares the same transport systems with arginine, and if the lysine levels are elevated, it can reduce the uptake of arginine (27, 28). Moreover, ornithine and arginine were downregulated in the 3- and 2-year-old fruiting bodies, which are important intermediates in the urea cycle, and it is hypothesized that this indicates a reduction in the urea cycle. This result supports the hypothesis that the amino acids produced by protein catabolism tend to synthesize their own proteins or other secondary metabolites. The amino acids and their differential metabolites in the 2- and the 3-year-old groups were more similar, while the 3-year-old groups had more types of differential metabolites compared with the others. Interestingly, not all the components increased significantly with age. Compared with the control group, the levels of saccharopine and L-arginine decreased significantly with increasing fruiting body development, but the level of gamma-glutamyl-beta-aminopropiononitrile decreased, which was 0.94-fold in the 3- and 2-year-old fruiting bodies. Thus, it may be as its highest level during the early stage of growth and development, and it tends to be stable as the development of fruiting body development decreases. Gamma-glutamyl-beta-aminopropiononitrile is a non-protein L-alpha amino acid derived from L-glutamine that is associated with the action of beta-aminopropionitrile action and plays a role in mouse metabolism (29). *Cis-*4-hydroxy-D-proline and *trans-*4-hydroxy-L-proline are two isomeric forms of hydroxyproline (Hyp) (30). *Trans-*4-hydroxy-L-proline is an abundant active ingredient in the metabolic process of collagen when it is degraded by microorganisms (31). *Cis-*4-hydroxy-D-proline undergoes some series of oxidative metabolism to produce CO_2_, and glucose among other things and participates in various biochemical reactions in living organisms (32). *Cis-*4-hydroxy-D-proline was upregulated 1.52-fold in 1-year-old fruiting bodies compared to 2-year-old fruiting bodies, and interestingly, no difference in content was detected in the 3-year-old fruiting bodies. In addition, some new active components were also detected in the 3-year-old fruiting bodies. Up regulation of Se-methylselenocysteine (MSC) up to 2.36-fold. MSC is a naturally occurring selenium compound that has been used in clinical therapy (33), and cell culture models have demonstrated the ability to inhibit cancer cell proliferation and promote apoptosis (34). Blocking the S and G1 cycles in a mouse pancreatic cell line inhibits cell proliferation (35). The mechanism of action of MSC is that it can down-regulate the molecular target of tumor survival (36) and enhance the delivery ability of anti-tumor drugs (37). In addition, it is not toxic to its own cells (38), and may be one of the main components of Sanghuang for its anti-tumor function. MSC also has the ability to improve cognitive deficits due to Alzheimer’s disease (39). An important indicator of cellular senescence evaluation is the decrease of glutathione content (40), which has the role of scavenging free radicals and maintains intracellular oxidative homeostasis (41), and its content is regulated by the intermediate dipeptide gamma-glutamylcysteine (γ-GC) (42). Some scholars believe that GSH cannot be synthesized ab initio in mitochondria and it is not the primary compound that exerts antioxidant homeostasis. The antioxidant activity of γ-GC can exert antioxidant effects to alleviate the damage of ROS on mitochondria (43). In addition, γ-GC has been used as a potential therapeutic agent for sepsis and found to reduce the inflammatory response and decrease the lethality of sepsis in studies on mice (44). The γ-GC content in the 3-year-old fruiting bodies was down regulated by 0.4-fold compared with the 1-year-old fruiting bodies. It has been reported that a variety of environmental factors can affect the content of amino acids in edible fungi, such as the cultivation mode, contents of substrates, and climatic and geographical conditions, particularly the temperature (24, 45). These changes that amino acid metabolism appeared to be essential during the maturation of fruiting bodies in this study.

Interestingly, the biosynthesis of plant secondary metabolites was the most noticeable among all metabolic pathways, including some amino acids, nucleotides, and other constituents. One important it was shikimic acid (SA), which was up regulated 16.89-fold in the 2-year-old fruiting bodies and 7.57-fold in 3-year-old fruiting bodies. SA is an essential substance in the anabolic pathway of hydroaromatic compounds and can be used as a viral inhibitor (46), in addition to its assembly into different bioactive substances that play an important role in anti-tumor (47), anti-inflammatory (48), and anti-platelet binding (49), and can be used in biopharmaceutical production, SA can also be used in the preparation of cosmetic preparations capable of exfoliating, whitening and moisturizing activities (50). Alpha-Linolenic (ALA), was significantly up regulated 1.95-fold in the 3-year-old fruiting bodies. ALA has a wide range of pharmacological effects (51), anti-metabolic function can be used in the treatment of obesity (52), cardiovascular disease (53–55), Piplartine is an alkaloid found in the Piper species plant (54) and has been used in traditional medicine for a long time (55). Its fruit is also used as a nutritional supplement. A variety of pharmacological activities including anticancer (56), cytotoxic (57, 58) and genotoxic abilities, (59) have been reported, especially for anticancer effects, which have been published in patents for clinical treatment. Piplartine exhibits anticancer activity in a variety of cells, relying on its toxic effects to kill cancer cells without causing damage to normal cells (60). Piplartine is expressed at high levels of expression in the perennial fruiting body of Sanghuang, suggesting it could be an essential component in the pharmacological activity of this organism and for the physiological functions of Sanghuang. Further research could explore its potential therapeutic applications.

In this study, it was also found that the years of growth that induced the quality of fruiting bodies were closely related to arachidonic acid metabolism. More interestingly, among these metabolites, the presence of 535-fold and 1,320-fold changes for 15-keto-prostaglandin F2α (15-keto-PGF2α) in samples B and C compared with samples A, respectively, those were detected greatly attracted our attention. Interestingly, almost no 15-keto-PGF2α was detected in the samples collected from the 1-year-old fruiting bodies, but its contents were extremely high in samples B and C, 15-keto-PGF2α is a metabolite of prostaglandin F2α (61). This result led to the hypothesis that 15-keto-PGF2α may be a functional component of the fruiting bodies of *S. vaninii*. It can also be used as a potential indicator to identify if the fruiting bodies have reached maturity and a standard to determine when they should be harvested. Thus, further study is needed to determine the pharmacological activity of *S. vaninii*. These compounds can be further isolated and purified to fully improve the medicinal value of *S. vaninii.* However, the small molecule compounds that correspond to the pharmacological activity of *S. vaninii* are still unclear and merit further study.

At different ages, 6-keto-prostaglandin F1α was only detected in fruiting bodies with a growth cycle of three years, with a fold of FC expression of 1.32 compared with 1-year-old samples and 1.22 compared with 2-year-old samples. 6-keto-prostaglandin F1α is the non-enzymatic hydrolysis product of prostacyclin (PGI2) and has therefore been used to measure the production of PGI2 (60). This compound has shown promise for the treatment of atherosclerotic and ischemic heart disease, In pathology, PGI2 and its analogs can inhibit myocardial hypertrophy and cardiac fibrosis induced by angiotensin II (Ang II) (62). In particular, the hypertrophic effect of PGF2α on cultured rat cardiomyocytes was not observed in mice owing to defective FP signaling. Injection of microgram levels of PGI into the brains of mice at normal physiological levels reduced their mean arterial pressure when they were injected with 6-keto-prostaglandin F1α (63), which causes an increase of blood pressure that may counteract the action of prostacyclin itself. Optimization of the growth stage selection process bears the potential of increasing the concentration of bioactive compounds in the extraction of subentities, ultimately enhancing the therapeutic efficacy of clinical treatments.

Secondary metabolites are chemical compounds that are produced by organisms but are unnecessary in the growth and development of the organism. The secondary metabolites of fungi are an important resource for important compounds in medicine, such as penicillin, rifampicin and anti-cholesterol compounds. A previous study demonstrated that some secondary metabolites, such as flavonoids and phenolic acids, have antioxidant activities and thus play an important role in anti-ageing. In this research, the levels of total and phenolic isoflavonoid compounds demonstrated a decreasing and increasing trend, respectively, as the growth time progressed (Table 2). Among these phenolic DAMs, the abundance of 1,2,3-trihydroxybenzene, 2-naphthol, catechol, hydroquinone were significantly higher in 2, and 3-year-old than those of control, implying that these metabolites may help to improve the antioxidant activity induced by the growth cycle to delay fruiting bodies senescence. Similar results were observed for isoflavonoids, such as formononetin, but biochanin was significantly lower in 2-, and 3-year-old than that of control. It was found that some terpenoids were observed for elevated in 2-, and 3-year-old fruiting bodies, including xanthoxin acid, abscisic alcohol, and 2-*trans*, 6-*trans-*Farnesal. The triterpenoids content was the highest in 3-year-old fruiting bodies. This result is consistent with previous research results.

In addition, our data showed that the activities of (4S, 5R)-4,5,6-trihydroxy-2-iminohexanoate and adenylosuccinic acid were remarkably increased in 2-, and 3-year samples. (4S, 5R)-4,5,6-trihydroxy-2-iminohexanoate is the parent compound of (4S, 5R)-4,5,6-trihydroxy-2-iminohexanoic acid, which is a hexonic acid derivative. It is dominant sources of variation, showing increases within growth age in each case, compared with the control. Adenylosuccinic acid is a purine ribonucleoside monophosphate and plays a role in nucleotide cycle metabolite, and can be converted into fumaric acid through adenylosuccinate lyase. This suggests that the energy metabolism was dominant during the maturation of *S. vaninii.*

## Conclusion

To our knowledge, this study is the first to report analyses of the substantial changes in *S. vaninii* owing to their age of growth at the metabolic level using TOF with UPLC–MS. Although many studies have reported that *S. vaninii* has pharmacological effects and significant biological activity, this research has usually been conducted on an isolated compound, which is studied without consideration of the other influences. The molecular mechanism of the pharmacological action of *S. vaninii* remains unclear, which affects its practical application and causes many obstacles. In this study, the analysis of the active components in the fruiting body of *S. vaninii* by metabolomics is helpful to understand the pharmacological activity, metabolic pathway and potential biological effects of *S. vaninii*, it is meaningful to reveal the regulatory mechanisms of metabolic pathways and the interactions between metabolites provides insights into physiological and pathological mechanisms, and it provides basic data to support for their further development and utilization. In addition, this study can provide the scientific basis for variety identification, quality control and evaluate the pharmacodynamics of *S. vaninii*.

## Data availability statement

The original contributions presented in the study are publicly available. This data can be found here: http://www.gpgenome.com/species/62345.

## Author contributions

CX performed the experiment, generated the data, and wrote the original draft. YaZ conceived and designed the experiments. ZL wrote some parts of the article and improved the article further. SZ provided the editorial support. JP and YuZ helped performing the experiment and analyzed the data. All the authors contributed to the article and approved the manuscript.

## References

[1] Kaushik A DeBerardinis R. Applications of metabolomics to study cancer metabolism. *Biochim Biophys Acta Rev Cancer.* (2018) 1870:2–14. 10.1016/j.bbcan.2018.04.009 29702206 PMC6193562

[2] Bracewell-Milnes T Saso S Abdalla H Nikolau D Norman-Taylor J Johnson M Metabolomics as a tool to identify biomarkers to predict and improve outcomes in reproductive medicine: A systematic review. *Hum Reprod Update.* (2017) 23:723–36. 10.1093/humupd/dmx023 29069503

[3] Zhang Y Li F Huang F Xie G Wei R Chen T Metabolomics analysis reveals variation in Schisandra chinensis metabolites from different origins. *J Sep Sci.* (2014) 37:731–7. 10.1002/jssc.201301242 24415683

[4] Brusco MJ. A comparison of simulated annealing algorithms for variable selection in principal component analysis and discriminant analysis. *Comput Stat Data Anal.* (2014) 77:38–53. 10.1016/j.csda.2014.03.001

[5] Wahyuni Y Ballester A Tikunov Y de Vos R Pelgrom K Maharijaya A Metabolomics and molecular marker analysis to explore pepper (Capsicum sp.) biodiversity. *Metabolomics.* (2013) 9:130–44. 10.1007/s11306-012-0432-6 23335867 PMC3548101

[6] Vagelos P Alberts A Martin D. Studies on the mechanism of activation of acetyl coenzyme A carboxylase by citrate. *J Biol Chem.* (1963) 238:533–40. 10.1016/S0021-9258(18)81295-9 13995702

[7] Wang H Ma J Zhou M Si J Cui B. Current advances and potential trends of the polysaccharides derived from medicinal mushrooms Sanghuang. *Front Microbiol.* (2022) 13:965934. 10.3389/fmicb.2022.965934 35992671 PMC9382022

[8] Wang H Ma J Wu D Gao N Si J Cui B. Identifying bioactive ingredients and antioxidant activities of wild Sanghuangporus species of medicinal fungi. *J Fungi Basel Switz.* (2023) 9:242. 10.3390/jof9020242 36836356 PMC9959451

[9] Li Y Yin C. In Vitro anti-oxidation, hypoglycemic and hypouricemic effects analysis of extracts from Sanghuangporus vaninii. *Mod Food Sci Technol.* (2022) 38:71–80. 10.13982/j.mfst.1673-9078.2022.5.0956

[10] Nicholson J Lindon J Holmes E. “Metabonomics”: Understanding the metabolic responses of living systems to pathophysiological stimuli via multivariate statistical analysis of biological NMR spectroscopic data. *Xenobiotica Fate Foreign Compd Biol Syst.* (1999) 29:1181–9. 10.1080/004982599238047 10598751

[11] Hu W Guo W Meng A Sun Y Wang S Xie Z A metabolomic investigation into the effects of temperature on Streptococcus agalactiae from Nile tilapia (Oreochromis niloticus) based on UPLC-MS/MS. *Vet Microbiol.* (2017) 210:174–82. 10.1016/j.vetmic.2017.09.012 29103689

[12] Dong H Zhao X Cai M Gu H E H Li X Metabolomics analysis of morchella sp. from different geographical origins of China using UPLC-Q-TOF-MS. *Front Nutr.* (2022) 9:865531. 10.3389/fnut.2022.865531 35449541 PMC9016275

[13] Johnson C Ivanisevic J Siuzdak G. Metabolomics: Beyond biomarkers and towards mechanisms. *Nat Rev Mol Cell Biol.* (2016) 17:451–9. 10.1038/nrm.2016.25 26979502 PMC5729912

[14] Wei P He M Teng H Han G. Metabolomic analysis of the aqueous humor from patients with central retinal vein occlusion using UHPLC-MS/MS. *J Pharm Biomed Anal.* (2020) 188:113448. 10.1016/j.jpba.2020.113448 32622112

[15] Crupi P Genghi R Antonacci D. In-time and in-space tandem mass spectrometry to determine the metabolic profiling of flavonoids in a typical sweet cherry (Prunus avium L.) cultivar from Southern Italy. *J Mass Spectrom.* (2014) 49:1025–34. 10.1002/jms.3423 25303392

[16] Cao L Zhang Q Miao R Lin J Feng R Ni Y Application of omics technology in the research on edible fungi. *Curr Res Food Sci.* (2023) 6:100430. 10.1016/j.crfs.2022.100430 36605463 PMC9807862

[17] Jiang A Liu Y Liu R Ren A Ma H Shu L Integrated proteomics and metabolomics analysis provides insights into ganoderic acid biosynthesis in response to methyl jasmonate in Ganoderma Lucidum. *Int J Mol Sci.* (2019) 20:6116. 10.3390/ijms20246116 31817230 PMC6941157

[18] Satria D Tamrakar S Suhara H Kaneko S Shimizu K. Mass spectrometry-based untargeted metabolomics and α-glucosidase inhibitory activity of Lingzhi (Ganoderma lingzhi) during the developmental stages. *Molecules.* (2019) 24:2044. 10.3390/molecules24112044 31146329 PMC6600326

[19] Park Y Jung E Singh D Lee D Kim S Lee Y Spatial (cap & stipe) metabolomic variations affect functional components between brown and white beech mushrooms. *Food Res Int.* (2017) 102:544–52. 10.1016/j.foodres.2017.09.043 29195984

[20] Farag M Fathi D Shamma S Shawkat M Shalabi S El Seedi H Comparative metabolome classification of desert truffles Terfezia claveryi and Terfezia boudieri via its aroma and nutrients profile. *LWT.* (2021) 142:111046. 10.1016/j.lwt.2021.111046

[21] Want E Masson P Michopoulos F Wilson I Theodoridis G Plumb R Global metabolic profiling of animal and human tissues via UPLC-MS. *Nat Protoc.* (2013) 8:17–32. 10.1038/nprot.2012.135 23222455

[22] Mamluk R Chen D Greber Y Davis J Meidan R. Characterization of messenger ribonucleic acid expression for prostaglandin F2 alpha and luteinizing hormone receptors in various bovine luteal cell types. *Biol Reprod.* (1998) 58:849–56. 10.1095/biolreprod58.3.849 9510976

[23] Fu C Mao W Gao R Deng Y Gao L Wu J Prostaglandin F2α-PTGFR signaling promotes proliferation of endometrial epithelial cells of cattle through cell cycle regulation. *Anim Reprod Sci* (2020) 213:106276. 10.1016/j.anireprosci.2020.106276 31987327

[24] Liu J Chang M Meng J Feng C Wang Y. A comparative proteome approach reveals metabolic changes associated with flammulina velutipes mycelia in response to cold and light stress. *J Agric Food Chem.* (2018) 66:3716–25. 10.1021/acs.jafc.8b00383 29584419

[25] Zhang Y Chen Y Guo Y Ma Y Yang M Fu R Proteomics study on the changes in amino acid metabolism during broccoli senescence induced by elevated O2 storage. *Food Res Int Ott Ont.* (2022) 157:111418. 10.1016/j.foodres.2022.111418 35761664

[26] Li R Sun Z Zhao Y Li L Yang X Cen J Application of UHPLC-Q-TOF-MS/MS metabolomics approach to investigate the taste and nutrition changes in tilapia fillets treated with different thermal processing methods. *Food Chem.* (2021) 356:129737. 10.1016/j.foodchem.2021.129737 33836358

[27] Taylor E Beckmann M Villarreal-Ramos B Vordermeier H Hewinson G Rooke D. Metabolomic changes in naturally MAP-infected holstein-friesian heifers indicate immunologically related biochemical reprogramming. *Metabolites.* (2021) 11:727. 10.3390/metabo11110727 34822384 PMC8625860

[28] Alamri A Burzangi A Coats P Watson D. Untargeted metabolic profiling cell-based approach of pulmonary artery smooth muscle cells in response to high glucose and the effect of the antioxidant vitamins D and E. *Metabolites.* (2018) 8:87. 10.3390/metabo8040087 30513640 PMC6316736

[29] Miana M Galán M Martínez-Martínez E Varona S Jurado-López R Bausa-Miranda B The lysyl oxidase inhibitor β-aminopropionitrile reduces body weight gain and improves the metabolic profile in diet-induced obesity in rats. *Dis Model Mech.* (2015) 8:543–51. 10.1242/dmm.020107 26035864 PMC4457038

[30] Hu S He W Wu G. Hydroxyproline in animal metabolism, nutrition, and cell signaling. *Amino Acids.* (2022) 54:513–28. 10.1007/s00726-021-03056-x 34342708

[31] Hara R Kino K. Characterization of novel 2-oxoglutarate dependent dioxygenases converting l-proline to cis-4-hydroxy-l-proline. *Biochem Biophys Res Commun.* (2009) 379:882–6. 10.1016/j.bbrc.2008.12.158 19133227

[32] Dziewiatkowski D Hascall V Riolo R. Epimerization of trans-4-hydroxy-L-proline to cis-4-hydroxy-D-proline during acid hydrolysis of collagen. *Anal Biochem.* (1972) 49:550–8. 10.1016/0003-2697(72)90461-7 5082949

[33] Selvam A Szekerczés T Björnstedt S Razaghi A Björnstedt M. Chapter Two - Methods for accurate and reproducible studies of pharmacological effects of selenium in cancer. In: Weerapana E editor. *Methods in Enzymology. Selenoprotein Structure and Function.* Cambridge, MA: Academic Press (2022). p. 25–62. 10.1016/bs.mie.2021.10.019 35101213

[34] Zeng H Combs G. Selenium as an anticancer nutrient: Roles in cell proliferation and tumor cell invasion. *J Nutr Biochem.* (2008) 19:1–7. 10.1016/j.jnutbio.2007.02.005 17588734

[35] Thompson H Wilson A Lu J Singh M Jiang C Upadhyaya P Comparison of the effects of an organic and an inorganic form of selenium on a mammary carcinoma cell line. *Carcinogenesis.* (1994) 15:183–6. 10.1093/carcin/15.2.183 8313506

[36] Bhattacharya A. Methylselenocysteine - a promising antiangiogenic agent for overcoming drug delivery barriers in solid malignancies for therapeutic synergy with anticancer drugs. *Expert Opin Drug Deliv.* (2011) 8:749–63. 10.1517/17425247.2011.571672 21473705 PMC3111097

[37] Azrak R Cao S Pendyala L Durrani F Fakih M Combs G Efficacy of increasing the therapeutic index of irinotecan, plasma and tissue selenium concentrations is methylselenocysteine dose dependent. *Biochem Pharmacol.* (2007) 73:1280–7. 10.1016/j.bcp.2006.12.020 17239826 PMC2062575

[38] Hu Y Chen Y Zhang Y Zhou M Song X Zhang B The protective role of selenium on the toxicity of cisplatin-contained chemotherapy regimen in cancer patients. *Biol Trace Elem Res.* (1997) 56:331–41. 10.1007/BF02785304 9197929

[39] Du X Shi Q Zhao Y Xie Y Li X Liu Q Se-Methylselenocysteine (SMC) improves cognitive deficits by attenuating synaptic and metabolic abnormalities in Alzheimer’s mice model: A proteomic study. *ACS Chem Neurosci.* (2021) 12:1112–32. 10.1021/acschemneuro.0c00549 33689275

[40] Ferguson G Bridge W. Glutamate cysteine ligase and the age-related decline in cellular glutathione: The therapeutic potential of γ-glutamylcysteine. *Arch Biochem Biophys.* (2016) 593:12–23. 10.1016/j.abb.2016.01.017 26845022

[41] Lu S. Regulation of glutathione synthesis. *Mol Aspects Med.* (2009) 30:42–59. 10.1016/j.mam.2008.05.005 18601945 PMC2704241

[42] Anderson M Meister A. Transport and direct utilization of gamma-glutamylcyst(e)ine for glutathione synthesis. *Proc Natl Acad Sci U S A.* (1983) 80:707–11. 6572362 10.1073/pnas.80.3.707 PMC393448

[43] Quintana-Cabrera R Bolaños J. Glutathione and γ-glutamylcysteine in the antioxidant and survival functions of mitochondria. *Biochem Soc Trans.* (2013) 41:106–10. 10.1042/BST20120252 23356267

[44] Yang Y Li L Hang Q Fang Y Dong X Cao P γ-glutamylcysteine exhibits anti-inflammatory effects by increasing cellular glutathione level. *Redox Biol.* (2018) 20:157–66. 10.1016/j.redox.2018.09.019 30326393 PMC6197438

[45] Xu S Wang F Fu Y Li D Sun X Li C Effects of mixed agro-residues (corn crop waste) on lignin-degrading enzyme activities, growth, and quality of Lentinula edodes. *RSC Adv.* (2020) 10:9798–807. 10.1039/c9ra10405d 35498574 PMC9050232

[46] Catalina D Quiroz D Carmona B Bolívar F Escalante A. Current perspectives on applications of shikimic and aminoshikimic acids in pharmaceutical chemistry. *Res Rep Med Chem.* (2014) 4:35–46. 10.2147/RRMC.S46560

[47] Candeias N Assoah B Simeonov S. Production and synthetic modifications of shikimic acid. *Chem Rev.* (2018) 118:10458–550. 10.1021/acs.chemrev.8b00350 30350584

[48] Xing J Sun J You H Lv J Sun J Dong Y. Anti-inflammatory effect of 3,4-Oxo-isopropylidene-shikimic Acid on acetic acid-induced colitis in rats. *Inflammation.* (2012) 35:1872–9. 10.1007/s10753-012-9509-7 22829139

[49] Huang F Xiu Q Sun J Hong E. Anti-platelet and anti-thrombotic effects of triacetylshikimic acid in rats. *J Cardiovasc Pharmacol.* (2002) 39:262–70. 10.1097/00005344-200202000-00013 11791012

[50] Batory M Rotsztejn H. Shikimic acid in the light of current knowledge. *J Cosmet Dermatol.* (2022) 21:501–5. 10.1111/jocd.14136 33825313

[51] Yuan Q Xie F Huang W Hu M Yan Q Chen Z The review of alpha-linolenic acid: Sources, metabolism, and pharmacology. *Phytother Res.* (2022) 36:164–88. 10.1002/ptr.7295 34553434

[52] D’Angelo S Motti M Meccariello R. ω-3 and ω-6 Polyunsaturated fatty acids, obesity and cancer. *Nutrients.* (2020) 12:2751. 10.3390/nu12092751 32927614 PMC7551151

[53] Bassett C McCullough R Edel A Patenaude A LaVallee R Pierce G. The α-linolenic acid content of flaxseed can prevent the atherogenic effects of dietary trans fat. *Am J Physiol Heart Circ Physiol.* (2011) 301:H2220–6. 10.1152/ajpheart.00958.2010 21963840

[54] Bezerra D Pessoa C de Moraes M Saker-Neto N Silveira E Costa-Lotufo L. Overview of the therapeutic potential of piplartine (piperlongumine). *Eur J Pharm Sci.* (2013) 48:453–63. 10.1016/j.ejps.2012.12.003 23238172

[55] Zhang P Huang Q Hua Z. Advances in studies on pharmacological effects of piperlongumine. *Chin Tradit Herb Drugs.* (2012) 43:201–4.

[56] Chen W Zou P Zhao Z Weng Q Chen X Ying S Selective killing of gastric cancer cells by a small molecule via targeting TrxR1 and ROS-mediated ER stress activation. *Oncotarget.* (2016) 7:16593–609. 10.18632/oncotarget.7565 26919094 PMC4941337

[57] Makhov P Golovine K Teper E Kutikov A Mehrazin R Corcoran A Piperlongumine promotes autophagy via inhibition of Akt/mTOR signalling and mediates cancer cell death. *Br J Cancer.* (2014) 110:899–907. 10.1038/bjc.2013.810 24434432 PMC3929888

[58] Bokesch H Gardella R Rabe D Bottaro D Linehan W Mcmahon J New hypoxia inducible factor-2 inhibitory pyrrolinone alkaloid from roots and stems of Piper sarmentosum. *Chem Pharm Bull.* (2011) 59: 1178–9. 10.1248/cpb.59.1178 PMC3166232 21881266

[59] Bezerra D Castro F Alves A Pessoa C Moraes M Silveira E In vitro andin vivo antitumor effect of 5-FU combined with piplartine and piperine. *J Appl Toxicol.* (2008) 28:156–63. 10.1002/jat.1261 17541943

[60] Kelton J Blajchman M. Prostaglandin I2 (prostacyclin). *Can Med Assoc J.* (1980) 122:175–9. 6988063 PMC1801769

[61] Gupta A. A comparative study of methylergonovine and 15-methyl prostaglandin F2α in active management of third stage of labor. *Obstet Gynecol Sci.* (2013) 56:301–6. 10.5468/ogs.2013.56.5.301 24328019 PMC3784129

[62] Tirapelli C Bonaventura D Tirapelli L de Oliveira A. Mechanisms underlying the vascular actions of endothelin 1, angiotensin II and bradykinin in the rat carotid. *Pharmacology.* (2009) 84:111–26. 10.1159/000231974 19657221

[63] Pace-Asciak C. Novel eicosanoid pathways: The discovery of Prostacyclin/6-keto Prostaglandin F_1α_ and the hepoxilins. *Mol Neurobiol.* (2005) 32:19–26. 10.1385/MN:32:1:019 16077180

